# The Rapid Manufacture of Uniform Composite Multicellular-Biomaterial Micropellets, Their Assembly into Macroscopic Organized Tissues, and Potential Applications in Cartilage Tissue Engineering

**DOI:** 10.1371/journal.pone.0122250

**Published:** 2015-05-28

**Authors:** Betul Kul Babur, Mahboubeh Kabiri, Travis Jacob Klein, William B. Lott, Michael Robert Doran

**Affiliations:** 1 Stem Cell Therapies Laboratory, Institute of Health and Biomedical Innovation, Queensland University of Technology at the Translational Research Institute, Brisbane, Australia; 2 Department of Biotechnology, College of Science, University of Tehran, Tehran, Iran; 3 Cartilage Regeneration Laboratory, Institute of Health and Biomedical Innovation, Queensland University of Technology, Brisbane, Australia; 4 Mater Medical Research—University of Queensland, Brisbane, Australia; University of Wisconsin-Madison, UNITED STATES

## Abstract

We and others have published on the rapid manufacture of micropellet tissues, typically formed from 100–500 cells each. The micropellet geometry enhances cellular biological properties, and in many cases the micropellets can subsequently be utilized as building blocks to assemble complex macrotissues. Generally, micropellets are formed from cells alone, however when replicating matrix-rich tissues such as cartilage it would be ideal if matrix or biomaterials supplements could be incorporated directly into the micropellet during the manufacturing process. Herein we describe a method to efficiently incorporate donor cartilage matrix into tissue engineered cartilage micropellets. We lyophilized bovine cartilage matrix, and then shattered it into microscopic pieces having average dimensions < 10 μm diameter; we termed this microscopic donor matrix “cartilage dust (CD)”. Using a microwell platform, we show that ~0.83 μg CD can be rapidly and efficiently incorporated into single multicellular aggregates formed from 180 bone marrow mesenchymal stem/stromal cells (MSC) each. The microwell platform enabled the rapid manufacture of thousands of replica composite micropellets, with each micropellet having a material/CD core and a cellular surface. This micropellet organization enabled the rapid bulking up of the micropellet core matrix content, and left an adhesive cellular outer surface. This morphological organization enabled the ready assembly of the composite micropellets into macroscopic tissues. Generically, this is a versatile method that enables the rapid and uniform integration of biomaterials into multicellular micropellets that can then be used as tissue building blocks. In this study, the addition of CD resulted in an approximate 8-fold volume increase in the micropellets, with the donor matrix functioning to contribute to an increase in total cartilage matrix content. Composite micropellets were readily assembled into macroscopic cartilage tissues; the incorporation of CD enhanced tissue size and matrix content, but did not enhance chondrogenic gene expression.

## Introduction

Cartilage tissue lacks reliable self-repair. Consequently, cartilage injuries often further degenerate rather than healing spontaneously. The tendency to degenerate makes osteoarthritis (OA) the leading cause of pain and disability in developed nations [1–4]. Currently, the repair of osteoarthritic lesions is not possible and joint replacements are the only surgical interventions that successfully restore OA joint function [5]. However, surgical repair of acute cartilage injuries and delayed onset of OA is possible to a limited extent. A range of surgical methodologies has been developed and the most promising methods utilize cell-based tissue engineering approaches.

The two clinically approved tissue engineering methodologies are Autologous Chondrocyte Implantation (ACI) [6] and Matrix-Assisted Chondrocyte Implantation (MACI) [7]. In both ACI and MACI, autologous chondrocytes are isolated from a biopsy of a non-load bearing site of the damaged cartilage. The isolated chondrocytes are then expanded *ex vivo*, before being transplanted into the primary defect site beneath a periosteum membrane isolated from the patient’s tibia (ACI) or on a manufactured type I/III collagen membrane (MACI). In both cases, the initial repair tissue lacks any cartilage matrix and is extremely fragile; typically the repaired joint is protected from full weight bearing for 2–3 months [8–10].

The initial fragility of the ACI/MACI repair tissue is related to the lack of mature cartilage ECM at the time of implantation. To overcome this problem a number of groups have explored strategies that involve the direct incorporation of mature cartilage matrix into engineered tissue. Peretti *et al*. assessed the bonding of 1 mm thick cartilage slices or chips with expanded chondrocytes; the bonding was successful however the repopulation of the cartilage pieces was inefficient [11–13]. The dense ECM network in donor cartilage matrix prevents effective cell infiltration, and as a result the repopulation of the 1 mm thick cartilage pieces was only successful on the superficial or in regions adjacent to outer exposed areas [11–13]. Using a refined approach Gong *et al*. combined thinner (10–30 μm) sections of donor cartilage and chondrocytes [14]. They reported that the repopulation of the cartilage sections was significantly enhanced in 10 μm thick sections even relative to the 30 μm thick sections [14]. This body of work indicated that donor cartilage matrix might not be efficiently repopulated unless used in units with dimensions approaching 10 μm in thickness. This characteristic suggests that unlike tissues such as skin, large pieces of donor cartilage matrix cannot be used to provide a template for the effective generation of larger 3D tissue structures.

An alternative to using large pieces of donor cartilage to provide macroscopic structure is the incorporation of cartilage matrix particles into scaffolds formed via conventional scaffold manufacture. Yang *et al*. described the fabrication of a natural porous ECM derived scaffold made of physically crushed, lyophilized and cross-linked native cartilage tissue [15]. Similarly, Zheng *et al*. compared porous scaffolds made of PLGA versus porous scaffolds made of pulverized cartilage versus a composition of both [16]. They identified the composite scaffold as optimal, and suggested that this was related to the combination of biomimetic natural nanofibrous cartilage pieces and the mechanically strong PLGA component [16]. Additionally, Shin *et al*. used a freezer mill to crush porcine cartilage pieces, then cross-linked the particles to obtain a porous scaffold which was then seeded with chondrocytes for tracheal implantation in a rabbit model [17]. The use of donor matrix to enhance engineered cartilage tissue quality appears to be a rational and promising approach.

Our team has previously utilized micropellet cultures to generate cartilage-like tissue [18, 19]. Micropellets differ from conventional pellets, with micropellets typically having 100–1000 cells each, whilst macropellets typically have 100,000–500,000 cells each. Chondrogenesis appears to be enhanced and more homogeneous in micropellets, and this likely reflects the improved mass transport enabled by their smaller diameter [19]. Whilst chondrogenesis appears to be improved in the micropellet system, relative to conventional pellet cultures, the rapid formation of cartilage matrix equivalent to native tissue remains challenging. We reasoned that the incorporation of microparticles of donor matrix into micropellets would provide a mechanism to rapidly increase the cartilage-like matrix volume of the micropellets, and ultimately enhance the tissue quality.

Herein we describe the optimization of a method to incorporate lyophilized donor bovine matrix particles into microtissues or macrotissues formed from bone marrow-derived mesenchymal stem/stromal cells (MSC). Hereafter we call the cartilage matrix particles Cartilage Dust (CD). We contrasted the volume contribution and chondrogenic induction capacity enabled through the incorporation of CD into microtissues or macrotissues formed from 180 cells or 200,000 cells each, respectively. To demonstrate the potential utility of this concepts in tissue engineering applications, we used a high throughput microwell system to manufacture thousands of micropellets containing CD, and demonstrated that these tissues could be amalgamated into larger tissues in a manner that might have utility in the engineering of larger repair tissues or perhaps in direct cartilage defect filling.

## Materials and Methods

### Microwell fabrication and surface modification

In previous work we outlined detailed methods for microwell insert manufacture [18]. In brief; using deep reactive ion etching a silica wafer was etched to have a microwell pattern of 600 microwells/cm^2^. Individual microwells had dimensions of 360x360x180 μm (S1 Fig). Polydimethylsiloxane (PDMS) replica molding was used to generate a negative that was then heat pressed into a sheet of polystyrene. The resulting polystyrene mold was used to manufacture PDMS sheets of microwells surfaces, from which discs were punched out to use as inserts within 24-well plates [18]. The 2 cm^2^ discs containing microwells were fit and glued with PDMS into 24-well plates (S1 Fig). Each 24-well plate insert contained 1200 microwells. The plates with microwell inserts were sterilized for 1 hour in 70% ethanol then rinsed multiple times with sterile PBS. Before cells were seeded, the inserts were treated with 5% pluronic acid (Sigma) solution for 5 minutes to block protein adhesion and prevent cell attachment to the PDMS surface. Then, the inserts were rinsed again with PBS and the cells were seeded into the wells containing the microwell inserts.

### Human bone marrow MSC isolation and expansion

#### Ethics

Bone marrow aspirate was collected from the iliac crest of healthy donors with full informed written consent in all cases. Mater Health Services Human Research Ethics Committee approved the consent procedure and held the consent documents. All tissue samples were provided to the research team in a de-identified manner. Ethical approval for this research was granted through the Mater Health Services Human Research Ethics Committee (Ethics number: 1541A) and the Queensland University of Technology Ethics Committee in accordance with the Australian *National Statement on Ethical Conduct in Human Research*.

Bone marrow MSCs were isolated directly from bone marrow aspirates. The collected bone marrow aspirate was diluted 1:1 with PBS and underlayed with 12 mL Ficoll Paque Plus (GE healthcare). The solution was centrifuged at 535xg for 20 minutes. Interface cells were collected, washed and resuspended in low glucose DMEM (DMEM-LG, Invitrogen) with PS and 10% fetal bovine serum (FBS, Invitrogen), then seeded in tissue culture flasks (T175, Nunc). After 48 hours, the non-adherent cells were removed and the adherent cells were further cultured to 80% confluence with medium changes every 3–4 days.

MSCs were expanded in monolayer in DMEM-LG supplemented with 10% FBS and PS in an incubator with 2% O_2_ and 5% CO_2_ atmosphere at 37°C. The cells were dissociated via 5-minute incubation with 0.25% TrypLE (Invitrogen) at 37°C when they reached confluence. The cells dissociated from one flask were divided equally into four and then seeded into new flasks until passage 3, and then the cells were used in the described studies.

### Chondrogenic differentiation medium

Chondrogenic medium was composed of DMEM-HG with 110 μg/mL sodium pyruvate (Invitrogen), 10 ng/mL recombinant human Transforming Growth Factor β1 (TGF- β1, Peprotech), 10^–7^ M dexamethasone (Sigma), 200 μM ascorbic acid 2-phosphate (Sigma), 40 μg/mL L-proline (Sigma), 1% Insulin-Transferrin-Selenium-Ethanolamine (ITS-X, Invitrogen) and PS. The chondrogenic medium was changed 75% every second day, the collected media was stored at -20°C for sulfated glycosaminoglycan (sGAG) analysis.

### Cartilage dust preparation

Bovine knee articular cartilage (obtained from a local butcher, animals were ~12 month-old) was used to generate cartilage dust. The cartilage tissue was harvested aseptically from femoral condyle as thin sections. The collected tissue was washed three times with sterile phosphate-buffered saline (PBS, Invitrogen) containing 100 U/mL penicillin and 100 μg/mL streptomycin (PS, Invitrogen). The tissue was frozen at -80°C overnight then lyophilized (Christ Alpha 1–2 LDplus). The lyophilized tissue was aseptically pulverized into a powder using a marble pestle and mortar (10 cm inner diameter) by vigorous crushing for 30 minutes. The powdered cartilage was stored dry at -20°C until use. The powdered cartilage was suspended in high glucose Dulbecco’s modified Eagle’s medium (DMEM-HG, Invitrogen) with PS immediately prior to initiating cultures, and the wetted cartilage pieces were filtered using a 40 μm cell strainer (BD Falcon) to eliminate pieces larger than 40 μm. The filtered micron size fraction of the powdered cartilage, referred as cartilage dust (CD), was washed twice with DMEM-HG including PS to generate a concentrated CD solution (~20 mg/mL). A 50 μL sample of that solution was added to each CD-containing macro or micropellets delivering ~1 mg (dry weight) CD per culture. Monochrome phase contrast images of the CD on a glass slide were taken with a Nikon DS-Qi1Mc camera using ECLIPSE Ti microscope at 4X magnification (Nikon, Japan). Cartilage dust particle size was measured using Feret’s diameter (ImageJ, NIH, USA).

### Macropellet formation

Macropellets were formed in 15-mL tubes (BD Falcon) using the conventional method. MSC macropellets contained only 2.2 x 10^5^ MSCs in 1mL chondrogenic media in tubes. MSC+CD macropellets contained both 2.2 x 10^5^ MSCs in 1 mL chondrogenic media and 1 mg CD, described in previous section, in tubes. CD macropellets contained only 1 mg CD in 1 mL chondrogenic media in tubes. Then the tubes were centrifuged at 400xg for 5 minutes. The macropellets were cultured in a 2% O_2_ and 5% CO_2_ incubator at 37°C for 14 days with loosened lids to enable gas exchange.

### Micropellet formation

Micropellets of ~180 cells each (a total of 2.2x10^5^ cells in 1200 micropellets) were formed using microwell PDMS discs in 24-well plates (Nunc) (S1 Fig) [18]. Each disc contained 1200 microwells therefore a single macropellet described in previous section was equivalent and compared to 1200 micropellets. Similar to macropellets, MSC micropellets contained only 2.2 x 10^5^ MSCs in 1mL chondrogenic media in wells. MSC+CD micropellets contained both 2.2 x 10^5^ MSCs in 1mL chondrogenic media and 1mg CD in wells. CD micropellets contained only 1mg CD in 1mL chondrogenic media in wells. Then the plates were centrifuged at 400xg for 5 minutes. The micropellets were cultured in a 2% O_2_ and 5% CO_2_ incubator at 37°C for 14 days. Monochrome phase contrast images of the cultures at day 1 and 14 were taken with a Nikon DS-Qi1Mc camera using ECLIPSE Ti microscope (Nikon, Japan). The diameters of the micropellets were then estimated from the images using ImageJ software.

### Micropellet assembly

In the second part of the study, the micropellets were assembled at different time points. Day 0 assembly was equivalent to macropellet formation. The other cultures were initiated as micropellets and subsequently assembled after either day 4, 7, 10 or 14 of culture as discrete micropellets. The assembly process was achieved by dislodging the micropellets from the microwells, via pipette aspiration, and pelleting the micropellets in a 15-mL tube by centrifuging at 400xg for 5 minutes. The assembled micropellets were then cultured in a 2% O_2_ and 5% CO_2_ incubator at 37°C with loosened lids to enable gas exchange. The total duration of chondrogenic culture was 22 days for the assembled tissues in this study.

### Sulfated glycosaminoglycan (sGAG) and DNA quantification

Tissues were digested through the addition of 0.25 mg papain (Sigma) solution directly to each tube or well followed by overnight incubation at 60°C. The digest was used to quantify both the DNA and the sGAG in tissue constructs. The medium collected during the medium exchanges was analyzed to determine the quantity of secreted sGAG. 1,9 Dimethyl methylene blue zinc chloride double salt (DMB, Sigma) was used for sGAG quantification. Digests or culture medium were dispensed in clear 96-well plates (Nunc), then DMB dye was added and the signal was measured at 590 using a plate reader (MULTISKAN GO, Thermo Fischer). Shark chondroitin sulfate (Sigma) was used to generate a standard curve.

PicoGreen dsDNA Reagent and Kit (Invitrogen) was used to quantify the DNA content in the micropellet digests. The papain digest and the PicoGreen solution was mixed in a black 96-well plate (half-area, Costar) and measured in a plate reader (FLUOstar OMEGA, BMG Labtech) at an excitation and emission of 480 nm and 520 nm, respectively.

### Gene expression

Trizol (Invitrogen) was used to extract mRNA from pellets as per the manufacturer’s protocol. The RNA concentration was measured using spectrophotometer (Nanodrop 1000, Thermo Scientific). Complementary DNA (cDNA) was synthesized from the mRNA template using SuperScript III RT and oligo(dT)20 kit (Invitrogen) as per the manufacturer’s instructions. The quantitative polymerase chain reaction (qPCR) was performed using Platinum SYBR Green qPCR SuperMix-UDG kit (Invitrogen). The primers (5’ to 3’, Geneworks) listed in S1 Table were used.

The qPCR reactions mixes were aliquoted using a liquid handler (epMotion M5073, Eppendorf). SYBR Green master mix was dispensed into 384-well plates (Applied Biosystems) and combined with cDNA template. The qPCR reaction was performed using ViiA real time PCR system (Applied Biosystems). The qPCR reaction was initiated with a 2-minute 50°C hold, followed by a 3-minute 95°C hold, and then proceeded with 40 cycles of 15-second at 95°C, 30-second at 60°C. The qPCR results were analyzed using ΔCt method and the gene expression was normalized to the geometric mean of two housekeeping genes (cyclophilin A and glyceraldehyde 3-phosphate dehydrogenase (GAPDH)).

### Histology

Cultured tissues were fixed in 4% paraformaldehyde (PFA, Sigma) for 30 minutes. Then collected in micro centrifuge tubes and embedded in optimum cutting temperature compound (OCT, Tissue-Tek) and frozen at -20°C. Embedded frozen tissues were sectioned in 10 μm thickness using a cryostat (Leica) then the sections were adsorbed on poly-lysine glass slides (Thermo Fisher), dried at room temperature, stored at -20°C. Before staining, the slides were brought to room temperature and the sections were fixed with 4% PFA for 20 minutes then rinsed with PBS.

For alcian blue staining, the sections were covered with filtered 1% alcian blue (Sigma) dissolved in 3% acetic acid with pH of 2.5 for 10 minutes. Then the slides were rinsed with PBS until the excess alcian blue was removed from the slides. The sections were counter stained using 4’, 6 diamidino-2-phenylindole (DAPI, Sigma). The sections were imaged using a Nikon ECLIPSE Ti microscope and images were taken using a Nikon DS-Fi1 camera. Alcian blue and DAPI images were merged using Photoshop (Adobe CS5) software.

For immunofluorescence (IF) staining, the slides were dried and borders were drawn around the sections using a hydrophobic PAP pen (Sigma). Then the sections were blocked using 3% goat serum (Invitrogen), 0.3% Triton X-100 (Sigma) in 1% BSA (Sigma) for 20 minutes at room temperature. Then the sections were incubated with primary antibodies for human collagen II and X both 1:100 dilution and raised in rabbit (Abcam) at 4°C overnight in a humidified chamber. Then the slides were washed twice with 0.3% Triton X-100 in PBS for 3 minutes, then rinsed once with PBS. Next, the sections were incubated with the secondary antibody (Cy-3 conjugated anti rabbit IgG, Abcam) diluted 1:500 in 1% BSA for 30 minutes at room temperature. The slides were washed twice with 0.3% Triton X-100 in PBS for 3 minutes, counter stained with DAPI then rinsed once with PBS. The sections were mounted using CC/Mount (Sigma) and cover slipped. The slides were imaged using an ECLIPSE Ti epifluorescent microscope (Nikon, Japan) and monochrome images were taken with a Nikon DS-Qi1Mc camera, and then colored using NIS Elements BR 3.2 software. The images were merged using Photoshop (Adobe CS5) software.

### Live imaging

A parallel 24-well plate containing MSC micropellets and MSC+CD micropellets was prepared and live imaging was utilized to track the assembly of the micropellets over the first 92 hours of culture. Medium was not changed during imaging and micropellets were cultured at 37°C under 5% CO_2_ and atmospheric oxygen instead of 2% O_2_. All live imaging was performed on a Live cell microscope (ZEISS, Germany), and image stacks were converted to AVI format using ImageJ software, then the videos were compressed and converted to mp4 using Movie Maker (Microsoft Windows, USA) software.

### Statistical analysis

All experiments were performed with *n = 4* biological replicates. Studies were repeated using 3 different donor MSCs. Data in graphs were represented as mean + standard deviation (SD). The significance was analyzed using SPSS software (SPSS Statistics 21, IBM, USA) and one-way analysis of variance (ANOVA) with Tukey’s post-hoc test was used to identify the statistical significance with a p-value smaller than 0.05. The significance was indicated using Roman numerals or symbols on the corresponding graphs, any two groups with same numerals or symbols were statistically equivalent and the groups not marked with the same numerals or symbols were statistically different.

## Results and Discussion

### Cartilage dust

**Fig 1:**
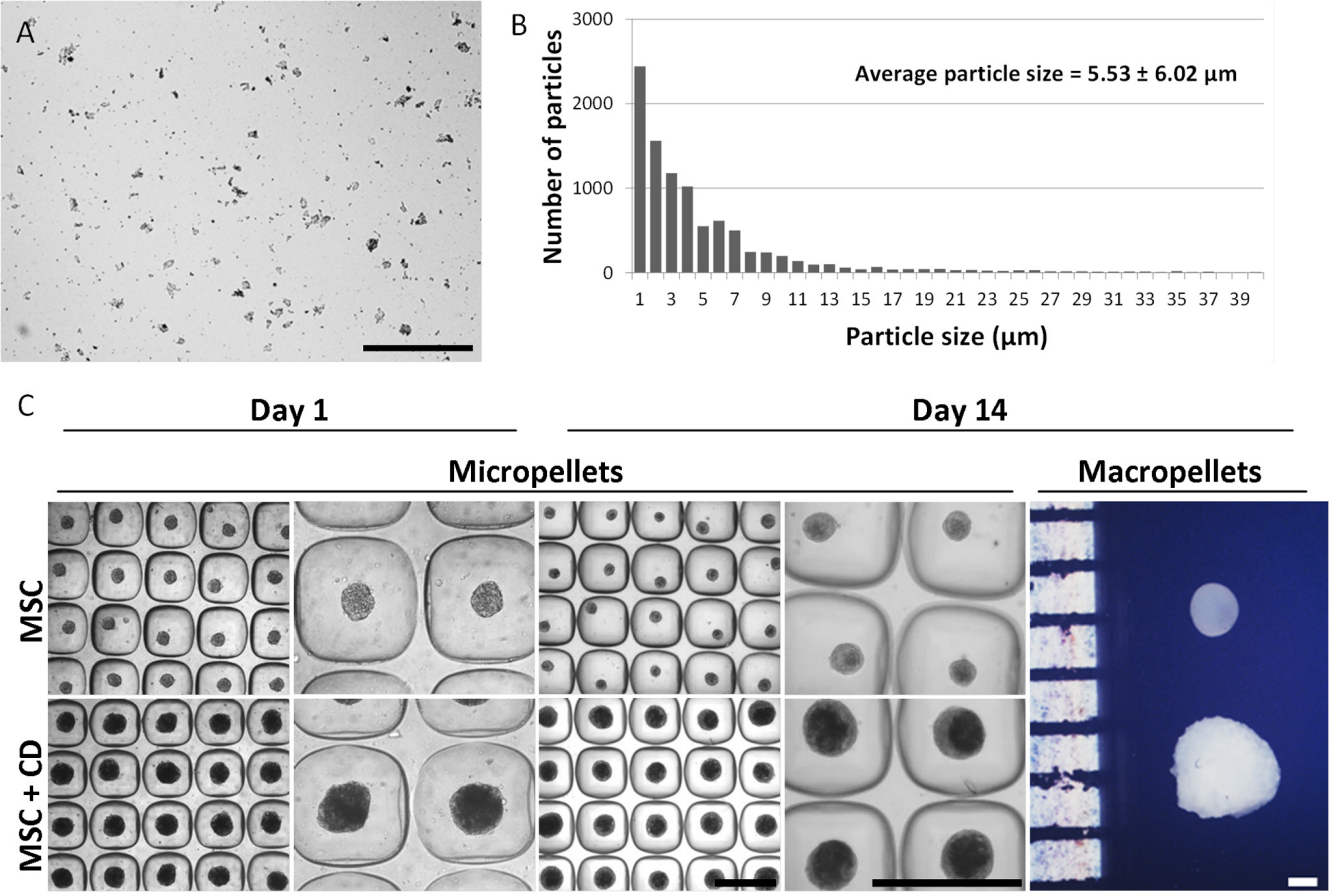
Cartilage dust (CD) characterization and micropellet morphology. Phase contrast image of CD demonstrated a gradient in particles size (A). The particles size distribution indicated that most of the particles had a size smaller than 10 μm and the average particle size was estimated to be 5.53 μm (B). Phase contrast images of MSC and MSC+CD micropellets on day 1 and 14 and macroscopic images of MSC and MSC+CD macropellets (C), the average diameters for MSC micropellets on day 1 was 100.8 ± 7.8 μm and on day 14 was 82.6 ± 10.8 μm, the average diameters for MSC+CD micropellets on day 1 was 176.1 ± 9.9 μm and on day 14 was 183.8 ± 16.2 μm Overall, MSC+CD macro and micropellets were greater in size. Abbreviations: MSC, cell only control; MSC+CD, composite (cell and cartilage dust). Scale bars: 500 μm.

CD particle size was first characterized via image analysis. A gradient in particle size was observed in cartilage dust (Fig 1A), and overall size distribution graph indicated that particle size was mainly less than 10 μm (Fig 1B). However even in low numbers, there were still particles greater than 10 μm since CD solution was filtered with a 40 μm cell strainer. In total 10,000 particles were characterized. The overall particle size average was estimated to be 5.53 ± 6.02 μm (Fig 1B) and the median particle size was 3.73 μm.

Devitalized xenogeneic matrix has been utilized in tissue engineering previously, but this particular approach has never been described. Here we pulverized lyophilized bovine cartilage and used only micron size particles, which provided efficient cell infiltration and repopulation of the donor matrix [20], since the size of the CD particles was in the same range with the mammalian cell size. The individual collagen fibers in CD particles might still be intact, however the overall collagen fiber organization was likely to be abolished during pulverization. Our pulverization method yielded different sizes of particles in CD (Fig 1A and 1B). The smaller particles tend to rapidly lose both the unwanted residual DNA as well as leach the desired sGAG molecules. The release of both DNA and sGAG increases proportionally to the increased surface area to volume ratio that occurs as the particle diameter is reduced. This phenomenon was noted nearly three decades ago; although at the time pulverizing cartilage into microparticles was done purposefully to enhance the extraction of sGAG from cartilage [21]. In our hands, it was necessary to store the CD dry and frozen to prevent loss of sGAG and only rinse it immediately before use to remove the bulk of the residual DNA.

### Pellet size

The addition of CD during the manufacturing of MSC+CD macro and micropellets always resulted in a significant increase in the pellet size, and this relative increase in size was maintained over the 14-day culture period (Fig 1C). The average diameters of the MSC micropellets and MSC+CD micropellets on day 1 were 100.8 ± 7.8 μm and 176.1 ± 9.9 μm, respectively. On day 14, the average diameters of the MSC micropellets and MSC+CD micropellets were 82.6 ± 10.8 μm and 183.8 ± 16.2 μm, respectively. The addition of CD to the tissue effectively doubled the diameter, significantly increasing the micropellet volume. The MSC micropellets were slightly smaller following 2 weeks of culture compared to day 1, whereas MSC+CD micropellets were slightly larger in size following 2 weeks of culture. MSC+CD micropellets appeared to have a darker core in phase contrast images at day 14 (Fig 1C). The CD appeared to be homogenously distributed within the MSC+CD micropellets at day 1, however CD was increasingly concentrated within the core of the micropellets throughout the culture (Fig 1C). MSC and MSC+CD micropellet formation was captured for the first 92 hours and the mechanism of MSC/CD self-assembly was documented in S1 Video and S2 Video. In videos, it is seen that the micropellet formation was completed within the first 4 hours, and that cell proliferation occurred at the periphery of the micropellets following micropellet assembly. Perhaps the most interesting outcome from this paper is the impressive mechanism by which the micropellet rolls around the microwell, attaches to the biomaterial/matrix, and then internalizes it. This feature may have potential utility in a number of tissue engineering and micropellet tissue mimic applications. It is foreseeable that this technology could readily be used to incorporate biomaterial particles that elute chondrogenic induction factors or other signal molecules. Alternatively, this approach could be used to rapidly incorporate bone matrix, for example, into micropellets to enable the rapid formation of bone tissue mimics for *in vitro* study or *in vivo* tissue repair.

The substantial volume increase in MSC+CD macro and micropellets supports the concept of using CD as an agent to rapidly increase the matrix content of engineered cartilage tissue. Utilizing CD, larger cartilage defects could be filled with reduced total number of cells. This would enable the rapid filling of defects with matrix-rich artificial tissue that had cell density and matrix composition similar to native cartilage tissue. A fortunate outcome of having the CD localize within the core is that the cellular surface of the MSC+CD micropellet makes individual micropellets very adhesive and readily assembled into larger tissues. Based on these observations, we reason that assembling MSC+CD micropellets will enhance the capacity of this concept to have utility in cartilage defect repair.

### DNA and sGAG production

**Fig 2:**
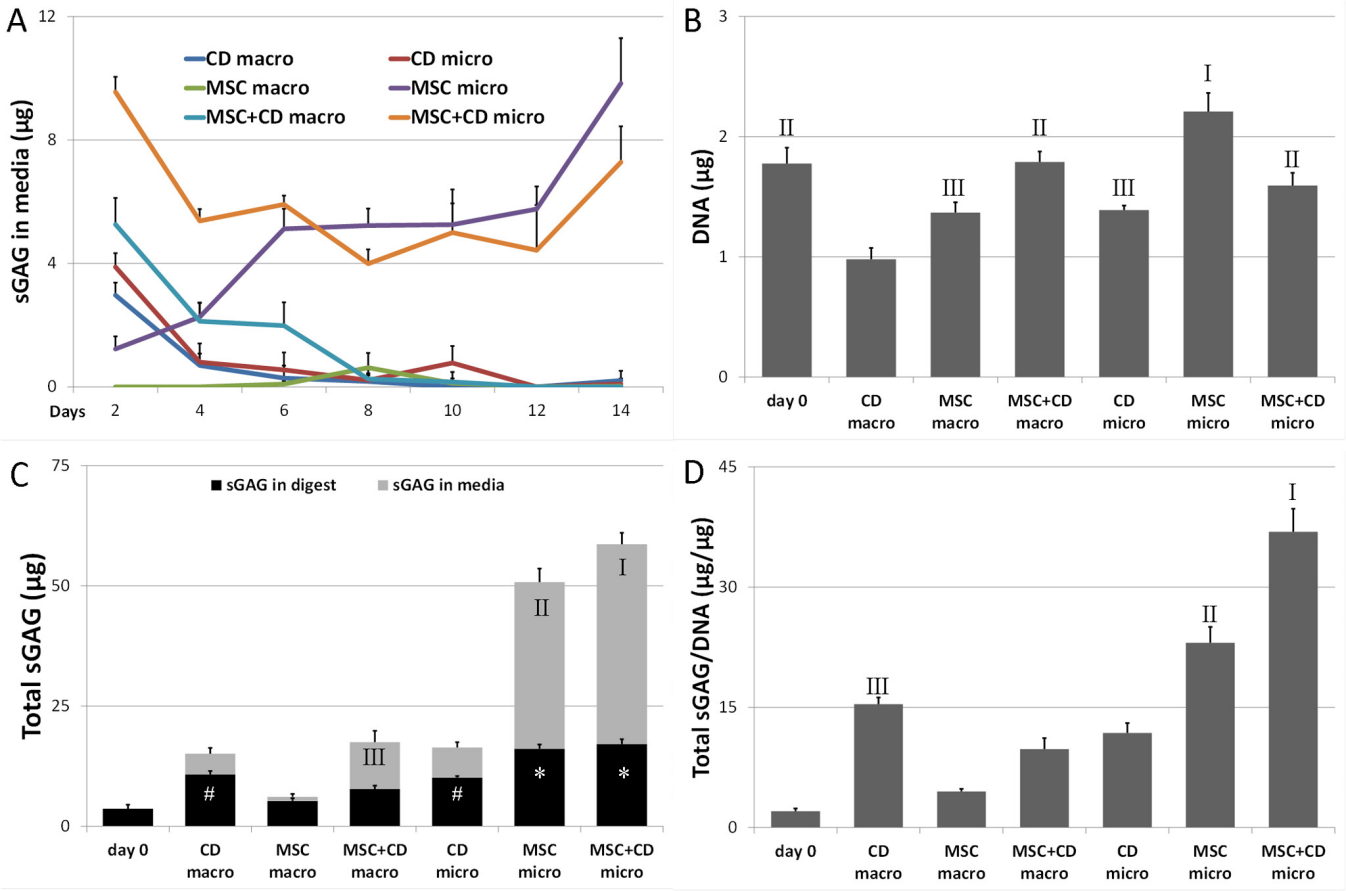
DNA quantification and chondrogenic differentiation. The quantity of sGAG in the media throughout the culture was higher for micropellets in general (A). The quantity of DNA at day 14 was the greatest for MSC micropellets and residual DNA was detected in CD macro and micropellets (B). The overall sGAG quantification in digest and in media demonstrated that the greatest quantity of sGAG was contained and eluted by MSC and MSC+CD micropellets (C). Total sGAG/DNA was calculated by dividing the quantity of the total sGAG (in digest and media) by DNA and this ratio was the greatest for MSC+CD micropellets (D). Abbreviations: CD, cartilage dust only control; MSC, cell only control; MSC+CD, composite (cell and cartilage dust); macro, macropellet; micro, micropellet.

There was residual DNA retained within CD only control samples (Fig 2B). At day 14 MSC micropellets had increased total DNA compared to day 0, indicating some cell proliferation. By contrast, there was no increase in DNA content in the MSC+CD macro and micropellets when compared to day 0 (Fig 2B).

Some DNA is retained possibly in greater size CD particles. In previous work, using human-derived CD, we were able to exclusively generate particles less than 10 μm in size and the human material retained only trace quantities of residual DNA [20]. In this study, the bovine cartilage was less malleable and tended not to be as easily pulverized, resulting in greater retention of DNA. Residual DNA could be removed through DNAse treatment, however additional processing of the CD would have also depleted sGAG content and chondrogenic cues therefore CD was minimally processed and not decellularized to maximize its biological potential on MSC chondrogenesis.

The quantity of sGAG measured in the media for the CD and MSC+CD macro and micropellets was greater during the early days of the culture (Fig 2A). The elution profiles from CD macro and micropellet controls indicate that the sGAG content in the medium over the first few days for MSC+CD macro and micropellets largely reflects elution from the CD rather than *de novo* sGAG synthesis by the MSC. Similarly, this time course also indicates that the sGAG elution from CD is completed by the end of the first week of culture (day 8). By contrast, MSC micropellets continuously increased their sGAG secretions over the first and second week of culture. It is clear that the sGAG secretion profile of the micropellets (MSC and MSC+CD) is superior to the macropellets (MSC and MSC+CD) in the second week of culture (Fig 2A).

Fig 2C outlines the total sGAG content in each of the tissue constructs. The sGAG values represent the actual quantity of sGAG produced by MSC and as well as the sGAG content that originated from CD. The calculations used to estimate these values accounted for the fact that we only performed a 75% volume medium exchange (rather than 100% exchange) every second day. CD macro and micropellets were able to retain the majority of their sGAG within the constructs while both MSC and MSC+CD micropellets eluted approximately twice as much sGAG as they retained (Fig 2C). Nevertheless, the overall sGAG retained and released to the media was significantly greater in micropellets relative to macropellets (Fig 2C). The loss of sGAG into bulk media only occurs during the micropellet culture and ceases when the micropellets are assembled (see “Micropellet assembly” section).

The total quantity of sGAG (both in media and in the tissue digest) from each sample was normalized to the total DNA. The greatest sGAG/DNA content was found in the MSC+CD micropellets followed by MSC micropellets (Fig 2D). The total sGAG content in these constructs was equivalent to the summation of the sGAG in the MSC micropellet plus the CD controls (Fig 2D). Previously published studies have reported synergistic benefits through the incorporation of donor cartilage matrix [22–24]. In our micropellet system the sGAG production is upregulated by 3–4 fold relative to the macropellet. It is possible that the increase in chondrogenesis resulting from the micropellet culture and TGFβ supplementation superseded the more subtle changes reported to be induced by the inclusion of donor matrix. Additionally, bovine-derived cartilage matrix was used in this study rather than human-derived cartilage matrix as described previously [20]. In our system, it appears that the increase in MSC+CD tissue volume and sGAG quantity represents the primary contribution of the CD to the cultures.

### Matrix deposition and distribution

**Fig 3:**
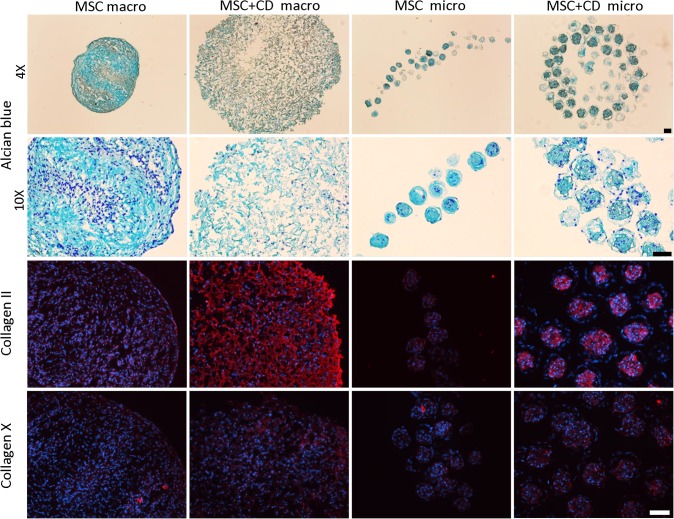
Histological assessment of MSC and MSC+CD macro and micropellets at day 14. The alcian blue staining demonstrated that MSC+CD macropellet was less intact when compared to MSC macropellet. Collagen II staining was stronger in MSC+CD macro and micropellets when compared to MSC macro and micropellets indicating that CD itself contained high quantity of collagen II. Collagen X staining was similar in all conditions. All the images were overlayed with DAPI staining. Abbreviations: MSC, cell only control; MSC+CD, composite (cell and cartilage dust); macro, macropellet; micro, micropellet. Scale bars: 100 μm.

Alcian blue staining demonstrated that all macro and micropellets contained sGAG (Fig 3). MSC macropellets demonstrated a heterogeneous sGAG distribution where some parts of the pellets were more intensely stained than others, and DAPI staining revealed that the less stained areas had more concentrated nuclei than sGAG rich areas (Fig 3). MSC+CD macropellet had less intense staining, reduced number of nuclei and the tissue structure appeared to be less intact (Fig 3). The staining for MSC and MSC+CD micropellets appeared similar, although it was evident that MSC+CD micropellets had a greater overall diameter.

Collagen II immunolocalizing suggested that CD itself was rich in collagen II, and MSC+CD macro and micropellets stained intensely for collagen II (Fig 3). The antibody manufacturer indicates that their human anti-collagen II antibody cross-reacts with bovine collagen II and we expected to be able to visualize the contribution of the bovine collagen. MSC macro and micropellets also stained positively for collagen II, however the intensity of the staining was substantially lower than the MSC+CD macro and micropellets indicating that the increase in collagen II content is mainly provided by the CD incorporation rather than *de novo* collagen II biosynthesis. Collagen X staining was relatively weak and similar for all conditions (Fig 3). The legitimate next step in future studies is the biomechanical characterization of generated tissues in order to measure the additional mechanical strength provided by CD incorporation.

### Gene expression

**Fig 4:**
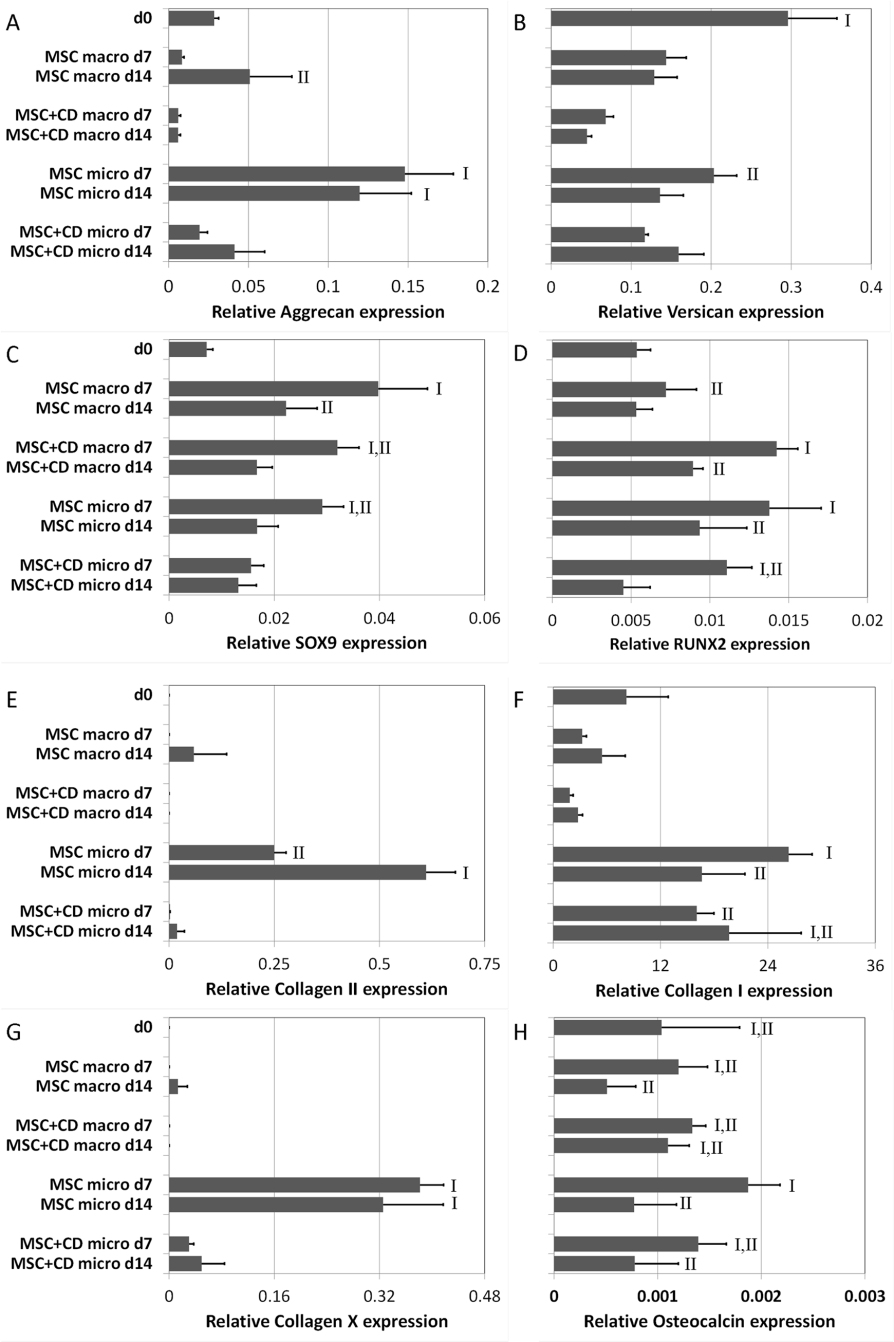
Gene expression analysis of MSC and MSC+CD macro and micropellets at day 7 and 14. Aggrecan (A), Versican (B), SOX9 (C), RUNX2 (D), Collagen II (E), Collagen I (F), Collagen X (G) and Osteocalcin (H) gene expressions were analyzed to assess the chondrogenic, hypertrophic and osteogenic characteristics of the generated macro and micropellets. Abbreviations: MSC, cell only control; MSC+CD, composite (cell and cartilage dust); macro, macropellet; micro, micropellet; d7, day 7; d14, day 14.

A was assessed for samples on day 0, day 7 and day 14 (Fig 4). Aggrecan expression was greatest in MSC micropellets on both days 7 and 14 (Fig 4A). Versican was significantly downregulated in all cultures relative to day 0, and the lowest expression was seen in the MSC+CD macropellets (Fig 4B). SOX9 was upregulated in MSC macropellets on day 7 (Fig 4C). RUNX2 was upregulated in MSC+CD macropellet and MSC micropellets on day 7 (Fig 4D). Collagen II expression was only upregulated in MSC micropellets on both day 7 and 14, being significantly higher on day 14 than on day 7 (Fig 4E). The low collagen II expression in MSC+CD micropellets also supports that the increase in collagen II content of these tissues (Fig 3) was mainly provided by the incorporation of CD. Collagen I expression was the lowest in MSC and MSC+CD macropellets (Fig 4F). The greatest relative collagen I expression was observed in MSC micropellets on day 7 (Fig 4F). Collagen X expression, similar to collagen II, was only upregulated in MSC micropellets on both day 7 and 14 (Fig 4G). Osteocalcin expression was the highest in MSC micropellets on day 7 but was significantly downregulated on day 14 (Fig 4H).

Overall the chondrogenic gene expression was not elevated in MSC+CD micropellets as much as it was in MSC micropellets confirming the previous results; no chondroinductive effects were observed as a result of CD supplementation (Fig 4). This observation suggests that chondrogenic factor supplement is superior to CD addition in inducing chondrogenic differentiation.

### Micropellet assembly

**Fig 5:**
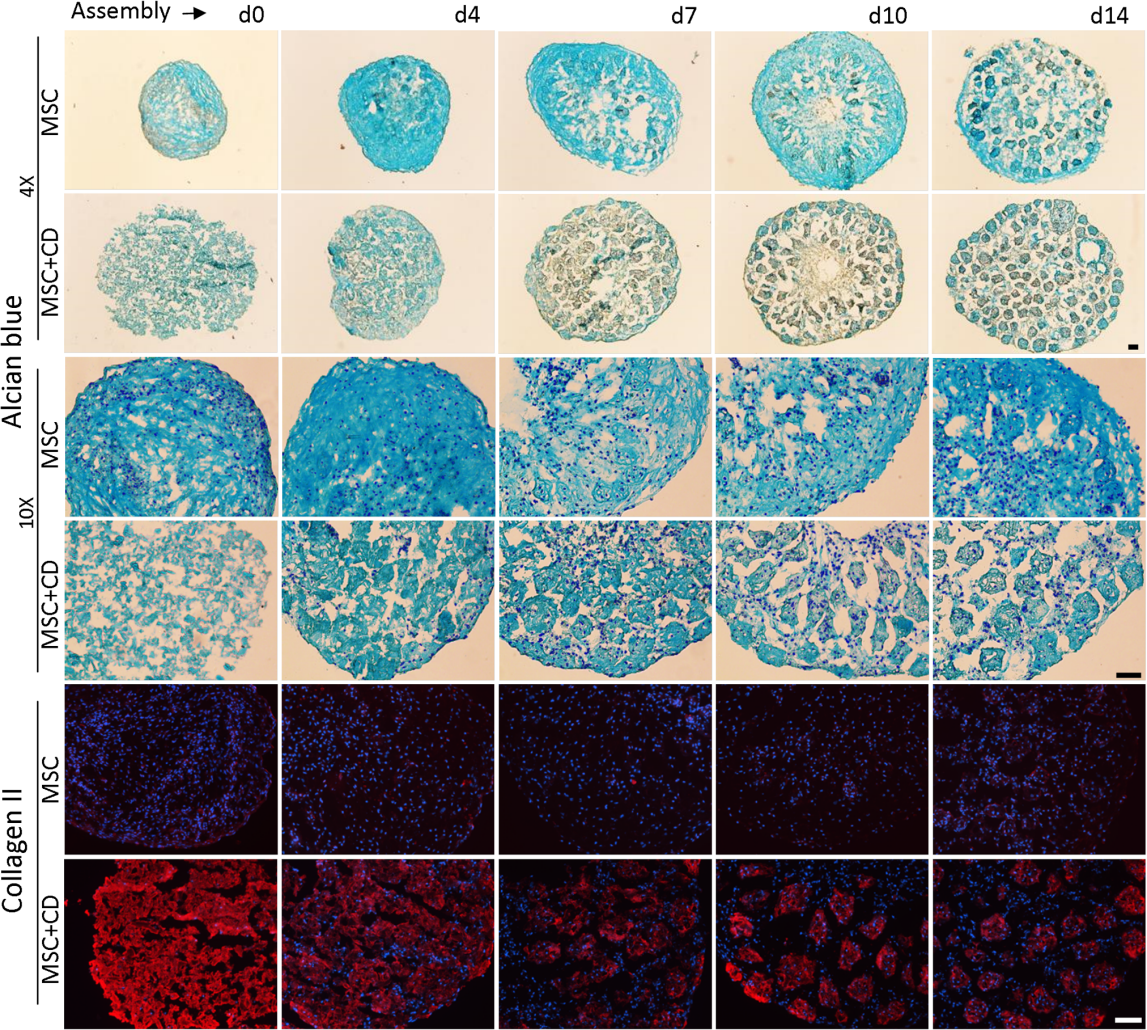
Histological assessment of the assembled tissues. Alcian blue staining showed inefficient integration of the CD particles in day 0 MSC+CD assembly (equivalent to MSC+CD macropellets) and decreasing integration efficiency was observed when the assembly day increased. Collagen II staining confirmed that the quantity of collagen II in MSC+CD assembled tissues was higher than the collagen II in MSC assembled tissues. Abbreviations: MSC, cell only control; MSC+CD, composite (cell and cartilage dust); d0, day 0; d4, day 4; d7, day 7; d10, day 10; d14, day 14. Scale bars: 100 μm.

Assembly of macropellets is previously utilized in order to engineer macroscopic cartilage tissues [25, 26]. Similarly in this study, the relative capacity of micropellets to be assembled into macroscopic tissues was compared at multiple time points in order to help predict the time point that would enable optimal tissue integration in defect repair applications. Specifically, MSC micropellets and MSC+CD micropellets were assembled into macrotissues following 4, 7, 10, or 14 days of culture as discrete micropellets. The assembled macrotissues were then matured in culture until day 22. Alcian blue staining revealed that MSC-only micropellets appeared to be more efficiently integrated into continuous and seamless macrotissues relative to MSC+CD micropellets (Fig 5). MSC+CD micropellet integration appeared to be more efficient at early time points (Fig 5). This is consistent with our previously published results, which demonstrated that the more mature a cartilage micropellet was, the less efficient it was at seamlessly integrating with other micropellet into a continuous macrotissue [18].

The collagen II staining confirmed the rich collagen II content of CD and if assembled at early time points, MSC+CD micropellets could form a collagen II rich cartilaginous tissue (Fig 5). Additionally, DAPI staining showed that few nuclei were observed for the day 0 MSC+CD assembly when compared to later assembly time points (Fig 5). Therefore, first generating composite MSC+CD micropellets then assembling them into macroscopic constructs can enhance revitalization of CD with MSC.

**Fig 6:**
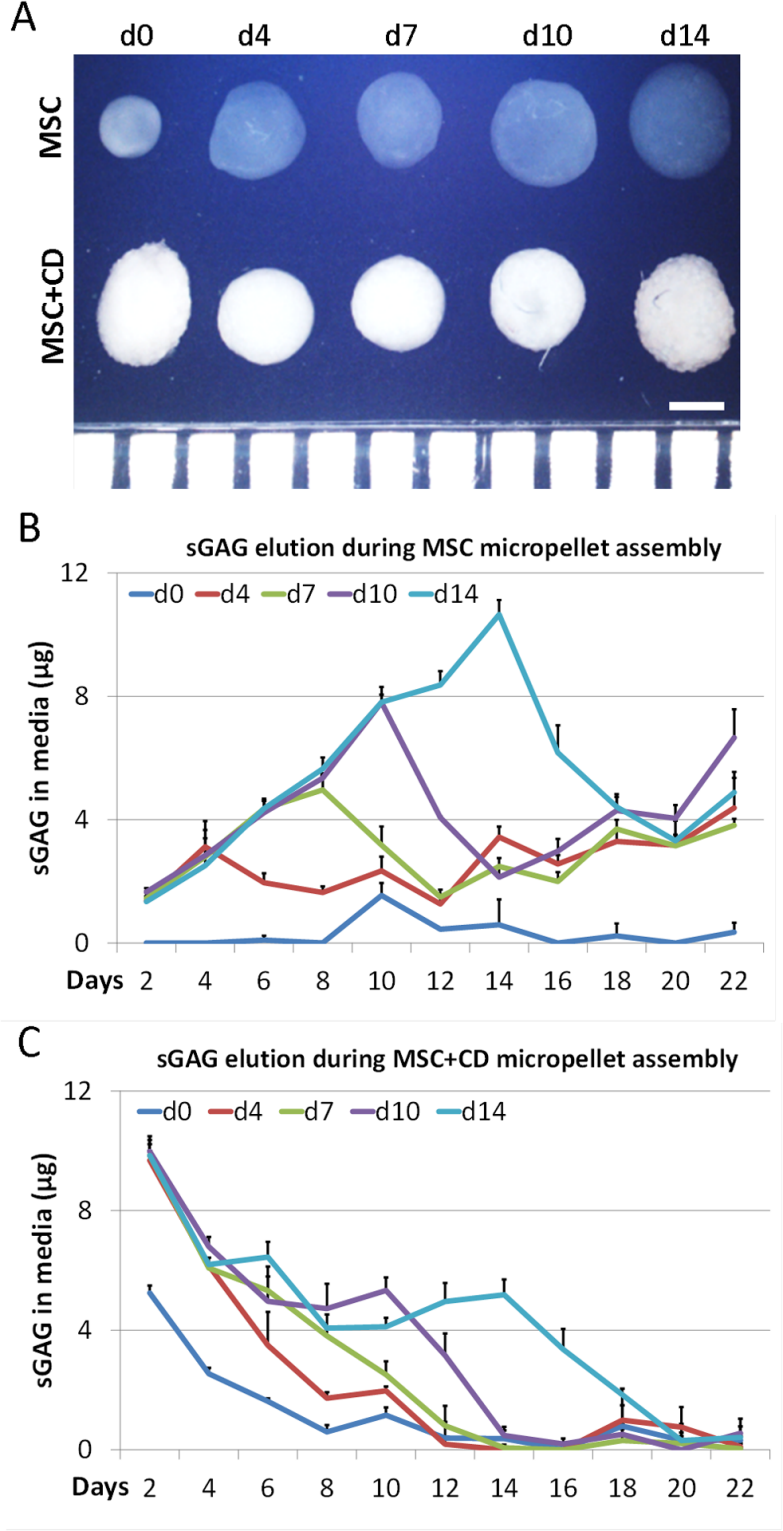
Morphology of the assembled tissue and quantification of the sGAG in media during assembly. MSC assembled tissues looked transparent whereas MSC+CD assembled tissues were more opaque and the day 0 MSC tissue was smaller than the rest of the assembled tissues. (A). The graph of sGAG in media during assembly of the MSC micropellets showed that the sGAG release to the media was diminished after assembly time points (B). The sGAG in media for the MSC+CD assembled tissues showed a decreasing trend in the first week, which is mostly the sGAG originating from CD (C). Abbreviations: MSC, cell only control; MSC+CD, composite (cell and cartilage dust); d0, day 0; d4, day 4; d7, day 7; d10, day 10; d14, day 14. Scale bar: 1 mm.

The macroscopic appearance indicated that MSC+CD assembled tissues were more opaque while the MSC assembled tissues appeared more transparent (Fig 6A). Day 0 assembled MSC tissue was noticeably smaller than the other assembled MSC tissues (Fig 6A). This observation may indicate that MSC micropellets can rapidly increase their size resulting in a greater total tissue volume relative to tissues formed immediately through the pelleting of a single cell suspension.

The quantity of sGAG released into the media was measured before and after the assembly of micropellets. Previously, it has been reported that the retention of sGAG is enhanced when chondrocyte macropellets are assembled into larger constructs [26]. Similarly, we observed that when MSC micropellets were assembled into macroscopic constructs, their sGAG elution slowed after the assembly time point (Fig 6B). This result is rational, as the surface area to volume ratio, from which the sGAG is eluted, decreases when larger tissues are formed. However elution pattern was different for MSC+CD micropellet assembly where a high quantity of sGAG was released to the media during the first week (Fig 6C), which suggest that the gradual sGAG loss was mainly originating from CD itself. Nevertheless, the elution of sGAG from both MSC and MSC+CD micropellets appears to be a transient event occurring during the early stage of discrete micropellet culture.

### Conclusion

Replicating native cartilage tissue ECM properties in tissue engineered cartilage remains a significant challenge in the field [27]. In approved therapies, such as ACI, chondrocytes are implanted into defects without the benefit of any previously established mature cartilage matrix. A cartilage defect site is a challenging microenvironment for tissue regeneration and expecting the rapid and efficient generation of functional repair tissue may not be rational, and thus successful cartilage defect repair strategies may require the combined use of donor and *de novo* cartilage matrix. Here we have described a novel strategy to incorporate mature donor cartilage matrix into engineered cartilage tissue. By supplying donor cartilage matrix in the form of microscopic cartilage dust (CD) we were able to overcome previously reported limitations in cell penetration and repopulation of larger dimension donor cartilage matrix pieces. To enable the uniform CD distribution in the engineered tissue, we first manufactured composite MSC+CD micropellets. Composite micropellets self-assembled into structures with a core of CD, and a cellular surface that facilitated the bridging of micropellets into macrotissues when they were in contact with each other. The logical next step in future studies is to assess biomechanical features of the generated tissues to further verify the benefits of utilizing CD in cartilage repair. Whilst the addition of CD did not enhance MSC chondrogenic differentiation, this delivery strategy resulted in uniform and rapid loading of donor cartilage matrix particles into MSC micropellets that may offer a mechanism to enhance cartilage defect repair. In a clinical setting, xenogeneic CD can be used as an off-the-shelf product, and the patient’s own bone marrow MSC can be harvested and expanded for two weeks, then MSC+CD micropellets can be manufactured within a week. Three weeks after the bone marrow harvest, the prepared micropellets can be injected into the defect site where CD will provide temporary mechanical support and MSC will contribute to *de novo* tissue regeneration. The exploitation of the described micropellet and biomaterial composite strategy may offer a unique template for the addition of other nano/microparticles capable of enhancing chondrogenesis through the release of growth factors or other signal molecules. Such strategies could enable control over cellular organization and the continued release of induction factors following implantation of the micropellets *in vitro*.

## Supporting Information

S1 Fig Schematic demonstrating the details of the microwell discs. (TIF)

S1 Dataset Spreadsheet containing all data presented in the figures. (XLSX)

S1 Table Primers used for gene expression analysis. (DOCX)

S1 Video First 92 hours of MSC micropellet formation. (MP4)

S2 Video First 92 hours of MSC+CD micropellet formation. (MP4)
