# Supplementary figures and images for: The Rapid Manufacture of Uniform Composite Multicellular-Biomaterial Micropellets, Their Assembly into Macroscopic Organized Tissues, and Potential Applications in Cartilage Tissue Engineering
Source: PLoS One. 2015 May 28;10(5):e0122250. doi: 10.1371/journal.pone.0122250 (PMC4447443; doi:10.1371/journal.pone.0122250)

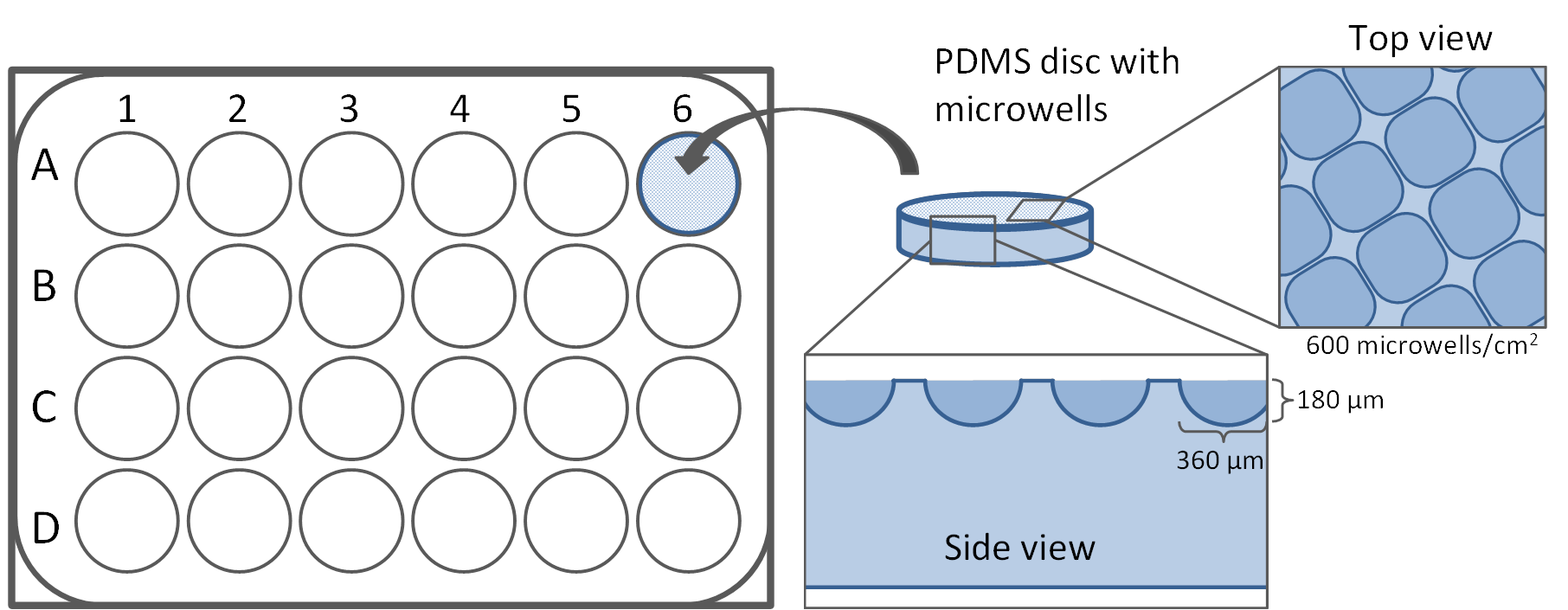

Supplement: S1 Fig — (TIF) [file pone.0122250.s001.tif]
